# The Structural Order of Crystallin Proteins During Early Human Lens Development

**DOI:** 10.1167/iovs.67.1.50

**Published:** 2026-01-23

**Authors:** Kiranjit K. Bains, James Bell, Robert D. Young, Qian Ma, Sally Hayes, Laura Howard, Olga Shebanova, Nick J. Terrill, Keith M. Meek, Justyn W. Regini, Andrew J. Quantock

**Affiliations:** 1 Structural Biophysics Group, School of Optometry and Vision Sciences, Cardiff University, Maindy Road, Cardiff, Wales, United Kingdom; 2 Diamond Light Source Ltd, Diamond House, Harwell Science & Innovation Campus, Didcot, Oxfordshire, United Kingdom

**Keywords:** lens development, crystallins, lens proteins

## Abstract

**Purpose:**

To study the structural arrangement of crystallin proteins in the human lens during development.

**Methods:**

Fetal human lenses were acquired from the UK Human Developmental Biology Resource and examined at four developmental stages; postconception weeks (pcw) 8 to 9 (*n* = 5), 12 to 13 (*n* = 3), 16 to 17 (*n* = 6), and 20 to 21 (*n* = 3). Small-angle X-ray scattering patterns were obtained as raster scans across the entirety of each lens using a 0.1 nm-wavelength, synchrotron X-ray beam measuring 200 × 150 µm at the specimen. Analysis of each small-angle X-ray scattering pattern provided a measure of the average nearest neighbor spacing and the extent of spatial order in the crystallin protein array.

**Results:**

Crystallins in the lens center became compacted as development progressed, with the average spacing measuring 19.9 nm at 8 to 9 pcw, 19.6 nm at 12 to 13 pcw, 18.7 nm at 16 to 17 pcw, and 17.7 nm at 20 to 21 pcw. The spatial order of the crystallin proteins in the lens center also decreased with time as indicated by a parameter called the coherence distance, which measured 26.9 nm at 8 to 9 pcw, 24.7 nm at 12 to 13 pcw, 24.6 nm at 16 to 17 pcw, and 24.9 nm at 20 to 21 pcw. Spacing and spatial order were consistently higher at the lens periphery, compared with the center, at all developmental stages studied.

**Conclusions:**

Spatiotemporal modifications in the array of crystallin proteins occur as the human lens develops. These are perhaps reflective of a shift in the relative proportions of crystallin subtypes present and have potential implications for the lens's developing refractive index.

The development of the human lens begins in early embryogenesis, with the lens emerging from surface ectoderm. Thickening and invagination of the ectoderm forms a lens placode that later detaches to create a hollow lens vesicle.^1^^,^^2^ The early formation of the lens capsule begins around 5 to 6 weeks of gestation (equivalent to 3 to 4 postconception weeks [pcw]) and matures by approximately week 10 of gestation (or 8 pcw).^3^ As development progresses, the posterior cells of the lens vesicle elongate and differentiate into primary lens fiber cells, which form the embryonic lens nucleus.^4^ Unlike many other tissues, these initial lens fibers remain in place and are not shed, leading to a densely packed structure^5^ surrounded by lens fibers that are continuously laid down throughout life from the anterior epithelial cells at the lens equator, which undergo mitosis and elongate, pushing older fibers toward the center of the lens.^6^^–^^8^ This arrangement is essential for maintaining the lens's transparency and refractive properties, specifically the gradient refractive index (GRIN), which are crucial for focusing light onto the retina^7^ (Fig. 1). As the lens continues to grow and undergo age-related molecular changes throughout life, its flexibility gradually diminishes, leading to presbyopia, which typically presents between 40 and 50 years of age.^5^ In parallel, prolonged exposure to UV light, oxidation, and glycation can cause protein denaturation and the buildup of insoluble aggregates, ultimately leading to cataract formation and lens opacification.^9^

**Figure 1.**
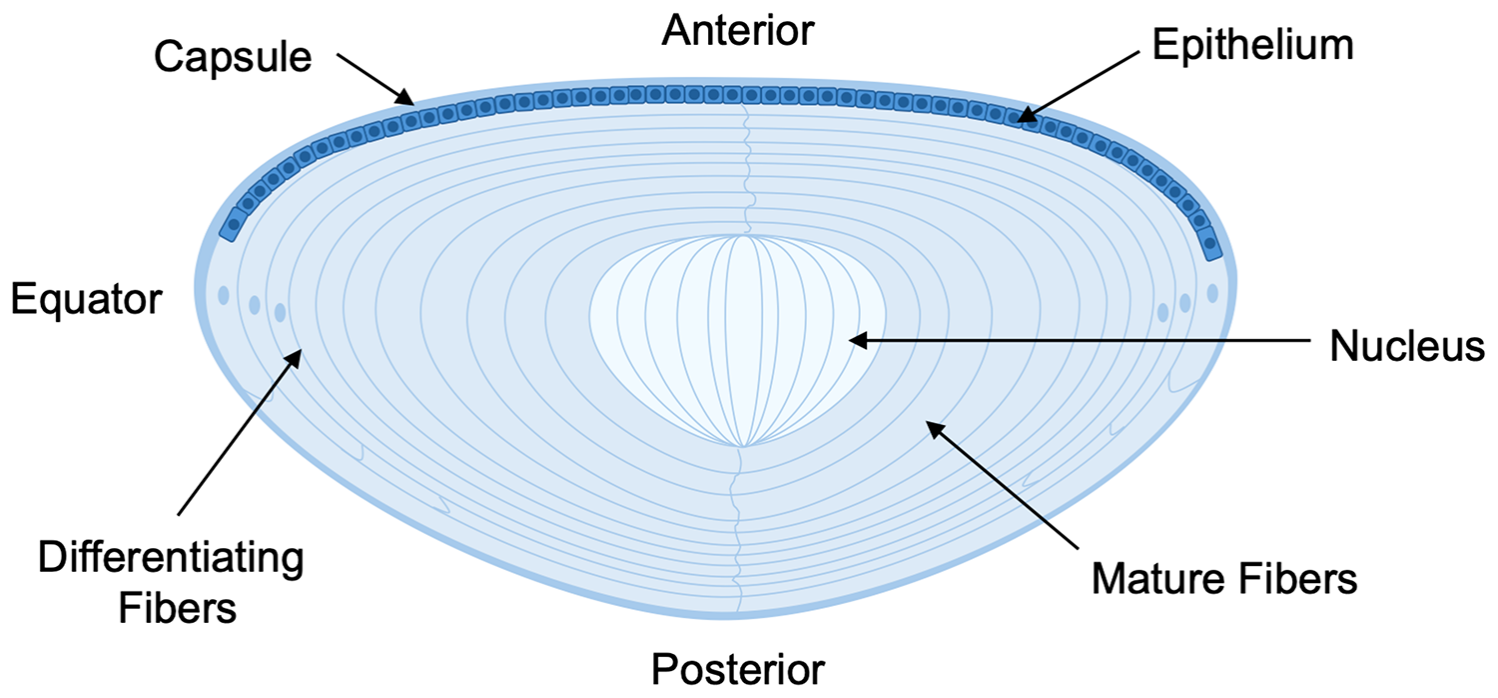
Cross-section schematic of a mature human lens.

Lens differentiation also involves specialized patterns of protein synthesis. The crystallin family of proteins, the most abundant proteins in the mammalian lens, which account for approximately 90% of its total water-soluble protein content, gradually accumulate during embryonic development and continue to increase throughout growth.^10^ The lens contains two fractions of water. The first fraction is free water, referred to as bulk water. The second is bound water, which is water that is bound to the proteins. In healthy adult human lenses, water accounts for 61.0% of the total mass, with 57.4% of the total water being bulk and 42.6% being bound.^11^ In humans, α-crystallin is the first crystallin to appear during lens differentiation, emerging between 4 and 8 gestation weeks (equivalent to 2 and 6 pcw), and belongs to a family of small heat shock proteins that function as molecular chaperones during early development.^12^^,^^13^ Chronologically, αB-crystallins emerge first in the lens placode, followed by the expression of both αA- and αB-crystallins in the lens vesicle and (later) lens epithelial and fiber cells.^12^ β- and γ-Crystallins are thought to play structural roles in the lens, with β-crystallin expression appearing around the same time as that of α-crystallin, while γ-crystallins emerge later in development, between 6 and 14 gestation weeks (equivalent to 4 and 12 pcw). Both β- and γ-crystallins are primarily expressed in lens fiber cells.^13^^,^^14^

Small-angle X-ray scattering (SAXS) has proven useful for the study of lens structure–function correlates ever since Delaye and Tardieu^15^ provided experimental evidence (based on SAXS patterns obtained from intact calf lenses and cytoplasmic extracts) in support of ideas that suggested that the short-range structural order of crystallin proteins within the lens underpinned its transparency.^16^^,^^17^ More recent work using X-rays from synchrotron sources has shown that α-crystallin (a small heat shock protein with chaperone-like capabilities) is able to maintain its structural integrity over a range of temperatures,^18^^,^^19^ with experiments based on SAXS and neutron scattering providing insights as to how various crystallins bind to target proteins and function as molecular chaparones.^20^^–^^22^ More widely across species, SAXS has also been used to identify a series of cell membrane complexes in the bisegmented cephalopod lens, which are believed to serve as channels for the internal movement of free water, ions, and other metabolic agents in a highly protein dense tissue,^23^ and to show, via studies of the lenses of fish sourced from lakes near structurally breached nuclear reactors in Chernobyl, Ukraine, and Fukushima, Japan, that significant exposure to environmental radiation does not lead to cataract formation and an alteration of crystallin structure.^24^ Using SAXS, the current study examines changes in crystallin spacing and structural order in early human lens development. Specifically, we trace how these parameters manifest themselves between 8 and 21 pcw, the broadest developmental window available for investigation. This approach affords valuable insights into the spatiotemporal dynamics of crystallin protein organization in the developing human lens, which might have implications for the development of a GRIN within the human lens.^7^

## Methods

### Tissue Collection

Donated eyes (*n* = 17) ranging from 8 to 21 pcw, were obtained from the MRC-Wellcome Trust Human Developmental Biology Resource (HDBR). Samples were snap frozen immediately after enucleation by HDBR before being transported to Cardiff University and stored at −80°C. Previous structural analyses of lenses using SAXS have shown that the X-ray scatter patterns are largely unaffected by the freeze–thaw process.^25^ Lenses were studied at four developmental stages pcw 8 to 9 (*n* = 5), pcw 12 to 13 (*n* = 3), pcw 16 to 17 (*n* = 6), and pcw 20 to 21 (*n* = 3). All procedures in this study were conducted in accordance with the Declaration of Helsinki and the ARVO guidelines on ethical research practices in ophthalmic and vision research. The UK National Research Ethics Service has given HDBR approval to function as a Research Tissue Bank (full details of the HDBR's ethics approval can be downloaded from www.hdbr.org/registration/ethics.html). The UK Human Tissue Authority also monitors how HDBR works to ensure it complies with the Human Tissue Act (2004).

### SAXS

Human fetal lenses were dissected from whole globes after thawing at room temperature immediately before imaging. SAXS patterns were collected on synchrotron beamline I22 at Diamond Light Source, the UK synchrotron (Oxfordshire, UK^26^). To prevent dehydration during SAXS data collection, the lenses were carefully wrapped in catering Clingfilm and placed within a custom 3D-printed sample holder. The holder was then placed on a motor-controlled three-axis stage within the beamline, ensuring that the center of the anterior lens surface was positioned perpendicular to the path of the X-ray beam. An optical in-line microscope was used for precision alignment of the sample and to ascertain the motorized stage coordinates corresponding with the outer edge of the sample.

To guard against radiation damage, prior to data collection the same region of a test fetal lens (16 pcw) was twice exposed to the X-ray beam, which resulted in the deterioration of the SAXS pattern and disappearance of the interference function at the second exposure. This was indicative of radiation damage caused by the initial X-ray exposure. Therefore, the X-ray beam was attenuated by placing a 50-µm-thick sheet of molybdenum (a second row transition metal and effective absorber of X-rays) in the path of the X-ray beam upstream of the specimen. This resulted in the X-ray flux being stepped down to such a level that three successive X-ray exposures through the same part of the lens gave rise to almost identical SAXS patterns (<1% loss of signal) with unaltered interference function positions and profiles (and thus unchanged Bragg spacings and coherence distances). Accordingly, the parameters used to achieve the three successive unchanged SAXS patterns were judged not to have caused any appreciable radiation damage to the crystallin protein array in the fetal lens tissue and were used throughout all experiments for data collection. Specifically, the X-ray beam was tuned to a wavelength of 0.1 nm and an elliptical profile of approximately 200 × 150 µm, with SAXS patterns recorded on an X-ray detector (Pilatus 2M, Dectris, Baden, Switzerland) positioned 6.25 m behind the sample. Data were captured by raster scanning the sample through the beam at 0.1- or 0.2-mm intervals (depending on the size of the lens) using 0.5-second exposures, ensuring the whole of each lens including a small buffer region were covered. SAXS patterns and subsequent measurements were calibrated using the 5.838-nm X-ray reflection of powdered silver behenate, a sample of which was mounted in the same sample holder as the studied lenses.

**Figure 2.**
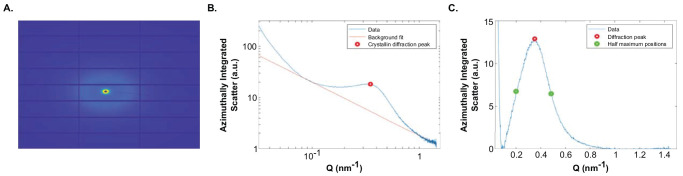
A typical SAXS pattern from fetal human lens (**A**), with radial integrations indicating X-ray scatter intensity vs. wavevector (Q) profiles before (**B**) and after (**C**) background subtraction.

Digitally recorded SAXS patterns were reduced using DAWN software^27^ (Data Analysis WorkbeNch, Harwell, UK) and processed using custom MATLAB code, which performed radial integrations to generate X-ray intensity (I) vs. wavevector (Q) profiles. Sample edges were identified by applying a threshold to the SAXS intensity at wavevector values known to be related to crystallin scattering. As shown in Figure 2, crystallin X-ray diffraction scattering features were separated from the Porod background by fitting and subtracting a power–law function using a minimization algorithm, before measurements were taken. The Bragg spacing (*d*), which for this study represents the average structural periodicity of the pseudo-lattice of crystallin proteins, was determined from the position of X-ray reflection peaks using the Bragg's equation:

(1) $$\begin{array}{c} n\lambda \;=2d\;sin\theta , \end{array}$$

where λ is the X-ray wavelength and θ is the Bragg angle. As we have shown previously in SAXS studies of both cornea and lens,^19^^,^^28^ here we approximate the extent of spatial order in the array of crystallin proteins by a parameter known as the coherence distance (*t*):

(2) $$\begin{array}{c} t\;\approx d\frac{2\theta }{\beta }, \end{array}$$

where *β* is the angular width of the X-ray reflection (i.e., its width at half height). Bragg spacing (*d*) and coherence distance (*t*) arising from the crystallin protein array were both derived from the SAXS patterns to examine variations in crystallin organization in the human fetal lens during early development. Specifically, changes in the crystallin packing structure were evaluated in the central one-third of each lens as well as along a transect through a midline corresponding with the tangential plane of the lens.

### Statistical Analysis

All values are reported as the mean ± the standard deviation of the mean. To compare the average central crystallin spacing and short-range ordering across early fetal development, normality and homogeneity of variances were assessed before performing a one-way ANOVA, followed by post hoc Tukey tests. A *P* value of <0.05 was considered statistically significant. All analyses were performed using JASP (version 0.19.3, Amsterdam, the Netherlands).

## Results

### SAXS

**Figure 3.**
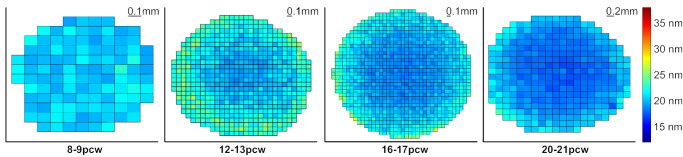
Color maps showing changes in the relative crystallin Bragg spacing (nm) in developing human fetal lenses. The images show representative data for each of the developmental groupings between 8 and 21 pcw.

**Figure 4.**
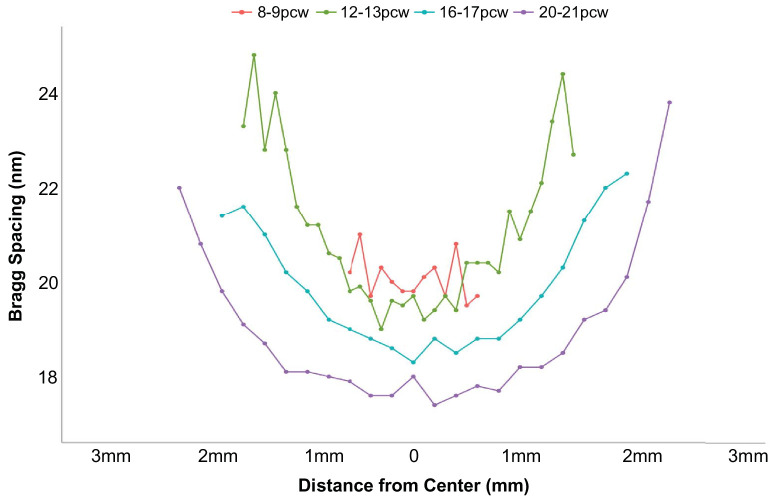
Vertical transects, along the midline of the lens, indicate average changes in Bragg spacing as development progresses.

**Figure 5.**
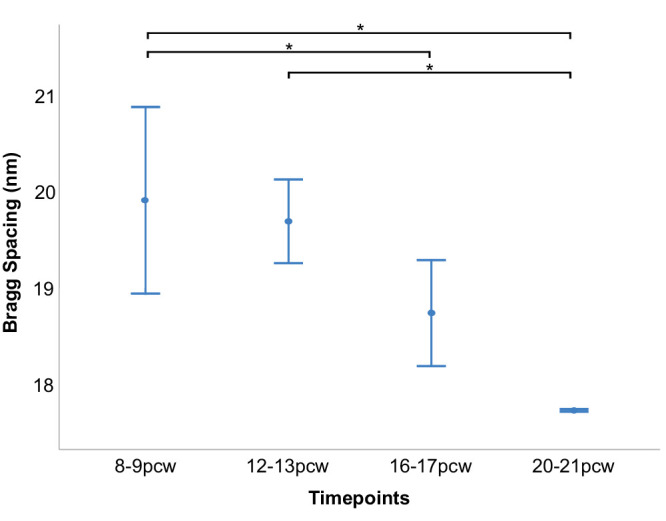
Average Bragg spacing measured at the centers of developing human fetal lenses. *P < 0.05.

SAXS raster scans across whole developing human lenses, with the X-ray beam passed front to back through the lens parallel to the path of the optical axis, allow us to calculate the average center-to-center spacing of crystallin proteins at multiple points, across each lens. It is important to note that the measured crystallin spacing values represent an average value throughout the full thickness of each lens at each point sampled by the X-ray beam. As illustrated in Figure 3, changes in crystallin spacing during lens development show that spacing between crystallins is fairly uniform across the lens at 8 to 9 pcw. However, as the tissue develops (12–21 pcw), the average crystallin spacings in the more peripheral regions of the tissue increase relative to the central region. This visual appreciation of the spatial variation of crystallin protein organization can be quantified by plots of Bragg spacing shown at 0.1 to 0.2-mm intervals across a central transect of each lens (Fig. 4). Although it was not possible to ascertain the precise anatomical orientation of the lenses during or after dissection, subsequent data analysis revealed the Bragg spacing values across each lens to be circularly symmetric (Fig. 3). As such, the transect findings, which showed evidence of greater compaction of crystallin proteins in the central region of developing human lenses, and the appearance of less closely spaced crystallins in the peripheral region of lenses from 12 pcw onward, may be considered valid for a randomly directed transect across the lens. Closer investigation of data obtained from the center of each developing lens reveals a decrease in average crystallin protein spacing between 8 to 9 pcw and 20 to 21 pcw (Fig. 5). Specifically, no significant alteration in crystallin spacing occurs from 8 to 9 pcw to 12 to 13 pcw, where it measures around 20 nm, with a steady reduction seen thereafter to 16 to 17 and 20 to 21 pcw (Table 1). A post hoc analysis of the data (Table 2) reveals a significant decrease in Bragg spacing from 8 to 9 pcw to 16 to 17 pcw (*P* = 0.049), 8 to 9 pcw to 20 to 21 pcw (*P* = 0.003) and 12 to 13 pcw to 20 to 21 pcw (*P* = 0.014).

**Table 1.** Summary of Average Bragg Spacing (nm) for Each Central Lens Region at Each Developmental Timepoint

Timepoint (pcw)	*N*	Mean Bragg Spacing ± SD	CV
8–9	5	19.9 ± 0.9	0.05
12–13	3	19.6 ± 0.4	0.03
16–17	6	18.7 ± 0.01	4 × 10^−4^
20–21	3	17.7 ± 0.4	0.02

CV, coefficient of variation; SD, Standard Deviation.

**Table 2.** Post Hoc Comparison of Average Bragg Spacing (nm) Obtained for Each Central Lens Region Across Pairwise Developmental Timepoint Combinations

Timepoint (pcw)	Mean Difference	95% CI for Mean Difference	*P*_tukey_
12–13			
16–17	0.9	[−0.4 to 2.3]	0.229
20–21	1.9	[0.4 to 3.5]	0.014^*^
8–9	−0.2	[−1.7 to 1.2]	9.961
16–17			
20–21	1.0	[−0.4 to 2.4]	0.181
8–9	−1.2	[−2.4 to 0.01]	0.049^*^
20–21			
8–9	−2.2	[−3.6 to −0.8]	0.003^†^

* *P* < 0.05.

† *P* < 0.01.

**Figure 6.**
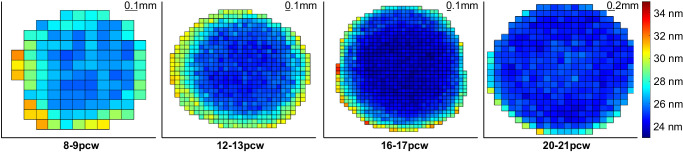
Contour maps showing changes in coherence distance (nm) in the developing human fetal lens. The images show representative data for each of the developmental groupings between 8 and 21 pcw.

**Figure 7.**
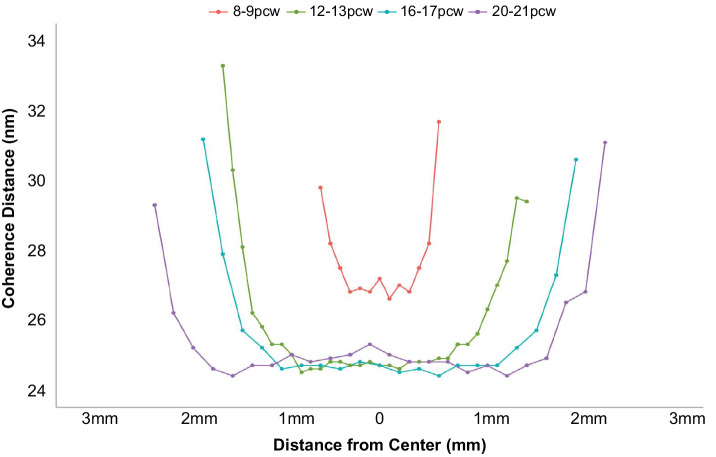
Vertical transects, along the midline, show changes in the coherence distance during human fetal lens development.

**Figure 8.**
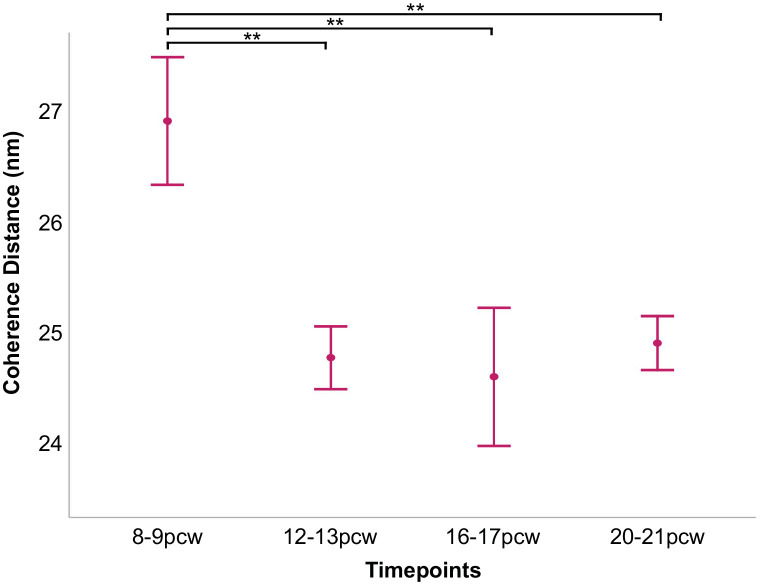
Average coherence distance in the centers of developing human fetal lenses. **P < 0.01.

The coherence distance is a measure of the length over which the structure remains ordered. It is sometimes referred to as the crystallite size and can be calculated from the width of the X-ray reflection. It follows from the relationship between t and β (Equation 2) that the narrower widths of the sharper X-ray reflections in SAXS patterns obtained from lenses are indicative of larger crystallite sizes than broader X-ray reflections. This might be caused by extended order in the array of specific crystallin proteins, but could also be reflective of the appearance of different crystallin subtypes as the lens develops. Figure 6 shows 2D maps of the coherence distance measurements obtained from four lenses representative of the developmental stage groupings, 8 to 9 pcw, 12 to 13 pcw, 16 to 17 pcw, and 20 to 21 pcw. This finding indicates that the degree of spatial order in the lenses is different in the peripheral tissue regions than in the central regions, and this is the case as early as 8 to 9 pcw. When the transect data are presented (Fig. 7), it can be seen that the change in spatial order does indeed occur in the peripheral regions of the lenses at all timepoints studied and that the change is quite abrupt over a relatively small region near the peripheral edges of the lenses, rather than a gradual change from the center outward. The indication from the data that the coherence distance is longer at 8 to 9 pcw compared with later developmental periods is confirmed by a more detailed interrogation of coherence distance measurements in the lens centers (Fig. 8). A statistical analysis of these (Tables 3, 4) reveals a significant lowering of the average coherence distance values from 8 to 9 pcw to 12 to 13 pcw, with stabilization thereafter to 16 to 17 pcw and 20 to 21 pcw. This is reflective of an altered spatial order in the array of crystallin proteins being attained by 12 to 13 pcw compared with 8 to 9 pcw, with a maintenance of organization to 20 to 21 pcw. When considering the main structural changes in the centers of the lenses identified in this series of SAXS experiments, we note that the average spacing of crystallin proteins remains constant between 8 to 9 pcw and 12 to 13 pcw and decreases thereafter (Fig. 5), whereas the coherence distance alters between 8 to 9 pcw and 12 to 13 pcw, and stabilizes thereafter (Fig. 8).

**Table 3.** Summary of Average Coherence Distance (nm) for Each Central Lens Region at Each Developmental Timepoint

Timepoints (pcw)	*n*	Mean ± SD	CV
8–9	5	26.9 ± 0.6	0.02
12–13	3	24.7 ± 0.3	0.01
16–17	6	24.6 ± 0.6	0.03
20–21	3	24.9 ± 0.2	0.01

**Table 4.** Post Hoc Comparison of Average Coherence Distance (nm) Obtained for Each Central Lens Region Across Pairwise Developmental Timepoint Combinations

Timepoints (pcw)	Mean Difference	95% CI for Mean Difference	*P*_tukey_
12–13			
16–17	0.2	[−0.9 to 1.3]	0.966
20–21	−0.1	[−1.4 to 1.1]	0.990
8–9	−2.1	[−3.3 to −1.0]	<0.001^*^
16–17			
20–21	−0.3	[−1.4 to 0.8]	0.845
8–9	−2.3	[−3.3 to −1.4]	<0.001^*^
20–21			
8–9	−2.0	[−3.1 to 0.9]	<0.001^*^

* *P* < 0.01.

## Discussion

Here, we use SAXS to identify and map spatiotemporal alterations in the structure and arrangement of crystallin proteins in developing human lenses. Suarez and associates previously used SAXS to study donated human lenses in the 5 to 82 year age range, reporting a consistent spatial organization of the crystallins up to the age of 55 years, with a diminution of the level of short range order thereafter.^29^ As in the current study, the spatial order of the crystallins was ascertained by an analysis of the first maximum of the interference function in the SAXS pattern, which is related to the reciprocal of the average diameter of a polydisperse system of interacting particles (i.e., the crystallins). Our previous analysis of SAXS patterns from intact rabbit lenses indicates that the average nearest neighbor spacing between the crystallin proteins indexes on a (Bragg) spacing of approximately 15 nm.^19^ The current analysis points to significant changes in the spatial organization of crystallin proteins in the human lens as fetal development proceeds. Specifically, at 8 to 9 pcw the lenses are small (approximately 1.5 mm across as judged by the horizontal span of the data points in Figure 3 and Figure 6 outside of which no tissue is encountered by the X-ray beam and no SAXS patterns recorded), and have a nearest neighbor spacing measured from the position of the interference function of approximately 20 nm (Table 1; Figs. 3, 5). This spacing does not change appreciably by 12 to 13 pcw, but subsequently decreases to less than 18 nm by 20 to 21 pcw in an approximate linear manner. The interpretation of this is the compaction of crystallin proteins with fetal age and lens growth.

As can be recognized from Figures 3, 4, 6, and 7 the diameter of the lens increases noticeably after 8 to 9 pcw to measure approximately 3 mm across at 12 to 13 pcw, just under 4 mm at 16 to 17 pcw, and approximately 4.5 mm by 20 to 21 pcw. The data that chart the Bragg spacing variation across a central transect of the lens (Fig. 4) at 8 to 9 pcw are a little noisy, which is perhaps reflective of the relative immaturity of the tissue. Thereafter, however, a clear trend emerges whereby the Bragg spacing that arises from the crystallin assemblies is lower in the central region of the lens than in more peripheral areas. It is well-established that the lens grows outwardly,^6^^–^^8^ with youngerlens fiber cells being laid down at the lens’ periphery. From the data presented here (Figs. 3, 4), it appears that a less dense arrangement of crystallin proteins is present in these outer fiber cells, with more compacted crystallin assemblies more centrally. This finding is consistent with an increase in the crystallin protein concentration from the periphery of the lens toward the central nucleus, noting that in the adult human lens, the crystallin concentration is approximately 450 mg/mL in the central nucleus and 200 mg/mL in the outer layers of the cortex.^30^ Perhaps the observation of a relatively high Bragg spacing peripherally may be related to the presence of different crystallin subtypes, with β-crystallin and γ-crystallin starting to appear alongside α-crystallin; however, this is speculation because SAXS is only able to identify the average spatial arrangement of the crystallins, not the specific protein subtype.

In a study of adult human lenses in the age range of 16 to 91 years, all specimens examined by synchrotron radiation Talbot interferometry were found to have a higher refractive index in the central nucleus than the more peripheral cortex.^31^ Indeed, this is the case in a range of species’ lenses (pigs, frogs, mice, newts, and fish), similarly examined.^34^ It is widely accepted that a GRIN within a lens bestows it with significantly beneficial optical properties.^7^^,^^33^ Crystallins, as the structural proteins of the lens, create the GRIN, which is linearly related to protein concentration—higher concentrations leading to a higher refractive index—and composition. Different crystallins (α, β, and γ), moreover, each have a different refractive index. It has been argued^7^^,^^33^ that, to attain the same optical performance as a GRIN lens, the focusing power of a lens with a homogeneous protein composition would require a substantially higher refractive index at all points within the lens, meaning that the required protein concentration would be unusually high, affecting the viscoelastic properties of the lens. The GRIN also helps to alleviate the problem of spherical aberration. With regard to fetal lens development, Pierscionek et al.,^34^ in studies of fetal bovine lenses, showed that, during the first half of the approximately 9-month gestation period, the refractive index of the fiber cell cytoplasm was relatively uniform. Then, beginning at approximately 4.5 months of gestation, a GRIN emerged, with higher refractive index values in the lens center and lower values more peripherally. GRINs have also been recorded in the lenses of 2-week-old mice,^35^ as well as in those of zebrafish 15-days post fertilization^36^ and chickens at embryonic day 10, approximately half-way to hatch.^37^ Spatiotemporal changes in the SAXS patterns in developing human lenses, reported here, imply that a GRIN is likely to be present by 12– to 3 pcw based on Bragg spacing measurements as indicators of crystallin protein concentration levels and refractive index.

Our data reveal that the coherence distance in the centers of the lenses decreases between 8 and 9 pcw and all later timepoints studied (Fig. 8). Of note here is the finding that the Bragg spacing and coherence distance in the lens center do not change hand-in-hand as the lens develops. Rather, there appears to be a lag of a few weeks in the structural reordering of crystallins within the developing human lens, whereby their short-range order changes (as indicated by the lowering of the coherence distance between 8 to 9 pcw and 12 to 13 pcw (Fig. 8) before they start to become more compacted (which occurs after 12–13 pcw) (Fig. 3). Where the Bragg spacings and coherence distances equate more closely is in their shared U-shaped profiles when transects across the lenses are examined (Figs. 4, 7). This finding reveals that the Bragg spacing and coherence distance are both proportionally higher in the lens periphery compared with the center. This situation is somewhat counterintuitive. One may expect that, as the crystallin protein concentration increases toward the central nucleus, the Bragg spacing would decrease and that this would then be accompanied by an increase in the coherence distance. This is based on the assumption that closer protein packing would lead to a higher degree of the ordering in the nearest neighbor periodicity of the pseudo-lattice of crystallin proteins. However, it must be noted that the lens is a structurally complex system, and not uniform. During a 2D scan of a lens, the X-ray beam passes through the whole thickness of the tissue and thus different regions, with X-ray scatter from regions of the lens with different protein concentrations all contributing to the final SAXS pattern. At the very periphery of the lens, the protein concentration is at its lowest, the bulk water at its highest and, of course, the lens is at its thinnest. It is in this region that the lens is structurally relatively uniform and at its simplest. Accordingly, the X-ray beam is only sampling one protein concentration. As the lens is traversed by the X-ray beam, it samples a greater number of different protein concentrations, as well as a decreasing amount of bulk water toward the central nucleus. As mentioned, the coherence distance is dependent on the intensity (i.e., peak height) and β, the angular width of the X-ray reflection of the interference function. In turn, the amount of scattered X-ray intensity of the interference function is dependent on a number of factors, including the electron contrast between the amount of bulk water and the electrons in the sample. Thus, at the lens periphery, with a relatively structurally uniform protein concentration and low electron mass due to the thinness of the lens at this location, the resultant inference function will be fairly sharp with a narrower angular width. As the X-ray beam samples regions with less bulk water, higher electron densities and a different number of GRIN zones, the resulting scattered X-ray peak will become broader and less intense. As can been seen in Figure 7, the coherence distance is greatest at the peripheral margins of the lens, decreasing inwardly. The values remain relative constant over much of the diameter of the lens for all weeks except 8–9 pcw. Interestingly, the coherence distance plots across the lenses mirror the human GRIN profiles in shape, with very steep sides and a flatter central portion,^32^ but are inverted. Animal GRIN profiles, in contrast, have a gentler slope toward lens center and are similar in appearance to a normal distribution curve.^33^ The data presented here, which chart the structural development of the fetal human lens, provide new insights into how crystallin proteins, as major and long-lived components of the lens, form.
